# Pharyngeal Detection of *Staphylococcus aureus* as a Possible Factor Related to Disgust Sensitivity in Humans

**DOI:** 10.3390/ijerph17218286

**Published:** 2020-11-09

**Authors:** Agnieszka Żelaźniewicz, Judyta Nowak-Kornicka, Renata Figura, Agata Groyecka-Bernard, Piotr Sorokowski, Bogusław Pawłowski

**Affiliations:** 1 Department of Human Biology, University of Wroclaw, 50-138 Wroclaw, Poland; judyta.nowak@uwr.edu.pl (J.N.-K.); renata.figura2015@gmail.com (R.F.); boguslaw.pawlowski@uwr.edu.pl (B.P.); 2 Institute of Psychology, University of Wroclaw, 50-529 Wroclaw, Poland; agata.groyecka@gmail.com (A.G.-B.); sorokowskipiotr@yahoo.co.uk (P.S.)

**Keywords:** prophylaxis, pathogen avoidance, health, behavioral flexibility, immunity, bacteria, asymptomatic colonization

## Abstract

Disgust triggers behavioral avoidance of pathogen-carrying and fitness-reducing agents. However, because of the cost involved, disgust sensitivity should be flexible, varying as a function of an individual’s immunity. Asymptomatic colonization with *Staphylococcus aureus* often results from weakened immunity and is a potential source of subsequent infections. In this study, we tested if pharyngeal colonization with *S. aureus*, evaluated based on a single swab collection, is related to an individual’s disgust sensitivity, measured with the Three Domain Disgust Scale. Levels of immunomodulating hormones (cortisol and testosterone), general health, and body adiposity were controlled. Women (*N* = 95), compared to men (*N* = 137), displayed higher sexual disgust sensitivity, but the difference between individuals with *S. aureus* and without *S. aureus* was significant only in men, providing support for prophylactic hypothesis, explaining inter-individual differences in disgust sensitivity. Men (but not women) burdened with asymptomatic *S. aureus* presence in pharynx exhibit higher pathogen disgust (*p* = 0.04) compared to individuals in which *S. aureus* was not detected. The positive relationship between the presence of the pathogen and sexual disgust was close to the statistical significance level (*p* = 0.06), and *S. aureus* colonization was not related with moral disgust domain.

## 1. Introduction

Disgust is a self-protective emotion, that evokes a negative effect toward potentially disease-bearing sources, triggering behavioral avoidance of pathogens and various fitness-reducing activities. Although behavioral repulsion, evoked by disgust, provides obvious advantages associated with infection avoidance [1,2], there is also a cost involved, such as dietary selectivity, loss of energy, and time devoted to disease avoidance. Thus, to be adaptive, the behavioral immune system should be flexible, varying in dependence of an individual’s immunity level and the risk of being infected with pathogens [2,3], and stronger aversive reactions should occur when the immunity is low, perceivers are more susceptible to infection, and the potential cost of infection is high [2].

Previous research showed that health and immunity level may explain the inter- and intra-individual differences in disgust sensitivity. For instance, women in the first trimester of pregnancy, when maternal immunity is suppressed, exhibit elevated disgust sensitivity [4]. Furthermore, longitudinal studies showed that changes in pathogen disgust sensitivity in menstrual cycle correspond to changes in immunovulnerability, that fluctuates within the cycle, following changes in immunomodulatory hormones levels, such as progesterone [5,6]; but see also for no result [2,7]. Additionally, cocaine-dependent individuals, whose immune functions are compromised, exhibit hypersensitivity to stimuli conveying a risk of infection and increase in the secretion of salivary IL-6 in response to the disgust provocation, possibly indicating an anticipatory mechanism to an imminent infectious challenge [8]. Furthermore, self-perceived health and self-reported frequency of infections has been shown to be negatively related with heightened contamination sensitivity [3,9] or with fear of disease-relevant invertebrates [10]. Recently pathogen avoidance has been also shown to be linked to stronger responses to the threat of COVID-19 [11]. Other studies showed that pathogen disgust sensitivity is negatively related with hand-grip strength, a simple measure of an individual’s general health [12]. Furthermore, disgust sensitivity has been shown to increase with age when the immune system weakens [13].

Furthermore, disgust sensitivity also influences social behavior and attitudes in situations involving some pathogen infection risk. Xenophobia (negative attitude toward others) and ethnocentrism (positive attitude toward own cultural in-group) seem to be positively related to the risk of disease in a given geographic area [14,15]. Additionally, individuals who score high on a measure of perceived vulnerability to disease were less likely to report having friends or acquaintances with disabilities and had stronger anti-immigrant attitudes toward immigrants from subjectively foreign locations [16,17].

Previous studies have shown that disgust sensitivity may even play a supportive role for physiological immunity. Disgust elevates core body temperature, pyrogenic cytokine such as TNF-α, and albumin levels, which are one of the first indicators of immune response activation [18,19]. Visual perception of other people’s symptoms of infectious disease causes white blood cells to produce higher levels of the proinflammatory cytokines, in response to stimulation by model bacteria. It suggests that merely visual perception of symptoms of infectious disease may cause more aggressive response of the immune system [20].

*Staphylococcus aureus* (*S. aureus*), widely distributed in the human body as a commensal bacterium, in some circumstances can act as a dangerous pathogen. The typical ecological niche of *S. aureus* in humans are anterior nares, but due to its virulence potential it is by far the most human pathogenic species in the genus *Staphylococcus*, and it can easily colonize many tissues and cause a variety of acute and chronic infections [21,22]. Although in asymptomatic long-term colonization, *S. aureus* goes largely unnoticed by a host due to a specific balance between pathogen virulence factors and host defence mechanisms [23], colonizing strains force immune system activation (e.g., induces expression of antibacterial peptides and cytokines) and serve as endogenous reservoir for many clinical infections [24]. During even local immunosuppression, infection caused by other pathogens, or when natural barriers are breached due to a skin cut, burns, or wounds after surgery, the pathogen can invade other tissues and cause systemic, potentially lethal infection [25]. More frequent autoinfections are observed in carriers whose health status and immune defenses are compromised, i.e., diabetic patients [26] or HIV-infected patients [27]. As *S. aureus* colonization often results from weakened immune defence mechanisms, and even asymptomatic colonization poses a constant threat as a source of subsequent infections and imposes physiological cost on an individual [28,29], one could expect that it may be linked with an increase in an individual’s disgust sensitivity.

Asymptomatic *S. aureus* colonization in the upper respiratory tract is very common in the human population [30]. Approximately 20% of healthy people almost always carry a strain. A large proportion of the population (c.a. 60%) harbors *S. aureus* intermittently and the strains change with varying frequency. Only a minority (20%) almost never carry *S. aureus* [30]. Research shows that there are several genetic factors predisposing for *S. aureus* carriage [23], and these genes are also responsible for effectiveness of an individual’s immune system [31], which may suggest a link between immunity and risk of bacterial colonization. Thus, in accordance with the prophylactic hypothesis, explaining variation in disgust sensitivity, one could expect that asymptomatic colonization with *S. aureus* may be positively linked to the level of an individual’s disgust sensitivity.

The aim of this study was to test if pharyngeal colonization with *Staphylococcus aureus* is related to an individual’s disgust sensitivity. We hypothesized that individuals colonized with *S. aureus* are characterized with higher disgust sensitivity, compared to noncolonized individuals. Although we were mainly interested in pathogen disgust, a component of disgust that has been suggested to evolve to motivate avoidance of traits or substances associated with disease-causing agents in ancestral environments [1], we additionally tested for the relationship between *S. aureus* colonization and sexual and moral disgust as well. These two components of disgust have been hypothesized to evolve to motivate avoidance of sexual partners and behaviors that would reduce one’s long-term reproductive success (sexual disgust: [2]), and avoiding individuals who inflict social costs on oneself or members of one’s social network (moral disgust: [32]). We hypothesized that individuals colonized with *S. aureus* are characterized with higher sexual and moral disgust sensitivity, compared to noncolonized ones. Furthermore, as disgust level may be influenced by some ongoing infections [33], we have controlled for inflammatory state, based on C-reactive protein (CRP) level. Additionally, as adiposity, cortisol, and testosterone modulate immune functions [34] and might be related either to *S. aureus* carriage [31] or disgust sensitivity [35], these factors were also controlled in the analyses.

## 2. Materials and Methods

### 2.1. Participants and General Procedure

Participants were recruited through information posted on social websites, information in local newspapers, and information in local pubs and bars. A total of 95 women (*M_age_* = 26.33, *SD_age_* = 2.73) and 137 men (*M_age_* = 27.97, *SD_age_* = 2.98) took part in the study. Men and women were selected for participation if they met the following criteria: no diagnosed chronic disease (diabetes, hypo/hyperthyroidism, autoimmune disorders, or metabolic problems) or hormonal disorders, not taking any hormonal medications, no current infections, and no recent use of antibiotics. Additional criteria for women were not using hormonal contraception and regular menstrual cycles (between 21 and 36 days). Additionally, women were recruited at the same moment of the menstrual cycle. They were asked to contact the research team at the 1st day of the menstrual cycle (self-assessed) and were invited to participate in the study between the 2nd and the 4th day of menstrual cycle (early follicular phase). The protocol used to recruit participants and collect data was approved by the Bioethics Commission at the Lower Silesian Chamber of Physicians and Dentists’ (HREC approval number 1/BO/2016). All participants read and signed the informed consent form for participation in the study and use of data for scientific purposes. The participants were informed in detail about all the procedures conducted in the study but they were not informed about the aims of the study or experimental hypotheses.

The study protocol consisted of taking throat swabs, fasting blood draw, body adiposity measurement, and answering a personal questionnaire. Body fat percentage (BFP) was measured in the fasting state, by bioimpedance using analyzer (Bodycomp MF, AKERN, Pontassieve, Italy) and computer software (BodyGram 1.2, Akern Bioresearch, Pontassieve, Italy). All procedures were performed on the same day, at early morning hours.

The general questionnaire, designed for this study, contained questions on factors of potential importance for disgust sensitivity level or probability of pharyngeal *S. aureus* detection. We inquired on demographic data (date of birth, place of living), education level and type of work, current and past health problems, cigarette smoking, and antibiotics use (Table 1). All participants declared no chronic or actual health problems, and none of the participants used antibiotics within the past few months. Individual’s disgust sensitivity or *S. aureus* prevalence were independent of the place of living, education level, smoking status, or type of work (*p* > 0.05).

### 2.2. Three Domain Disgust Questionnaire (TDDS)

Disgust sensitivity was measured with the Three Domain Disgust Scale (TDDS; [32]). The 21-item TDDS is a self-report instrument that measures disgust sensitivity separately for pathogen, sexual, and moral domains, each subscale containing a description of seven actions or statements. TDDS asks participants to rate the degree to which they find various concepts disgusting on a “0” (Not at all disgusting) to “6” (Extremely disgusting) seven-point Likert-type scale. The instructions for the questionnaire were: “The following items describe a variety of concepts. Please rate how disgusting you find the concepts described in the items, where 0 means that you do not find the concept disgusting at all, and 6 means that you find the concept extremely disgusting.” Examples of pathogen items include “Standing close to a person who has body odor” or “Seeing some mold on old leftovers in your refrigerator” (Cronbach’s α = 0.70). Examples of sexual disgust items include “Hearing two strangers having sex” or “A stranger of the opposite sex intentionally rubbing your thigh in an elevator” (Cronbach’s α = 0.81). Moral disgust items include “Stealing from a neighbor” or “Shoplifting a candy bar from a convenience store” (Cronbach’s α = 0.79).

### 2.3. S. aureus Isolation and Identification

Throat swab samples were taken from the posterior wall of the pharynx using transport swabs with enclosed tubes, containing sterile viscose tip swabs with AMIES transport medium (Deltalab, Barcelona, Spain). Up to 12 h from the collection, swabs were streaked onto Columbia agar with 5% sheep blood (blood agar) (Biocorp, Warsaw, Poland) and Mannitol Salt agar (MSA) (BioMerieux SA, Marcy l’Etoile, France). Both plates were incubated aerobically at 37 °C: 24 h for COS and 48 h for MSA. Plates were read after 1 or 2 days of incubation. Cream or yellow colony with β-hemolysis on blood agar were used in a further procedure to identify *S. aureus*. The yellow colonies on MSA, reflecting the strain’s ability to ferment of mannitol were identified as *S. aureus*. Single colonies from blood agar and MSA were also tested with latex slide agglutination test (Staphyloslide latex test kit, Becton Dickinson, Franklin Lakes, USA). *S. aureus* possesses clumping factor and/or protein A and can be easily differentiated using Staphyloslide^®^ (Becton Dickinson, Franklin Lakes, USA) test from other staphylococci. Positive results of *S. aureus* identification were based on both colony morphology and positive agglutination test. To minimize the observer bias, the samples were coded and blinded for the observer.

### 2.4. General Health Evaluation

Participants’ general health status was controlled with basic physiological parameters, commonly used in clinical practice. Blood morphology with smear consists of total leukocyte count (including each subpopulation e.g., neutrophils, lymphocytes, and monocytes fraction), red blood cells count, platelets count, hemoglobin level, and hematocrit. All participants had blood morphology parameters within the normal range or had one parameter slightly beyond the recommended standard, which in clinical practice is recognized as “healthy”. High sensitivity CRP level (hsCRP) was evaluated using commercial kits (catalogue number DE740011, Demeditec Diagnostics GmbH, Kiel, Germany). Test procedure was performed in accordance to instruction supplied with the kit. As few participants had hsCRP value slightly higher than the normal range (i.e., higher than 5 µg/mL), this variable was controlled in the statistical analyses.

### 2.5. Hormone Measurements

Serum cortisol and free testosterone (fT) concentrations were measured with enzyme-linked immunosorbent assay (ELISA) and commercial kits (catalogue number DE1887 and DE2924 respectively, Demeditec Diagnostics GmbH, Kiel, Germany). Serum samples were assayed in duplicate, according to manufacturer instructions supplied with the kit. Both within and between assay variability were less than 10% with the assay sensitivity of 2.5 ng/mL for cortisol and 0.06 pg/mL for fT. The standard curve was constructed by plotting the absorbance of each standard (vertical axis) against its concentration (horizontal axis). The concentration of each sample was calculated in relation to the standard curve and expressed in ng/mL for cortisol and in pg/mL for fT.

### 2.6. Statistical Analyses

As cortisol, testosterone, and hsCRP values were not distributed normally, logarithmic values were used in the statistical analyses.

The relationship between categorical variables (e.g., sex and *S. aureus* carriage) were analyzed with chi-squared tests. The difference in controlled variables (fT, cortisol, hsCRP, and body adiposity) and disgust level between sexes and between the individuals colonized and noncolonized with *S. aureus* were analyzed with t-test for independent variables. The relationships between continuous variables (disgust sensitivity and controlled variables) were tested using correlation coefficient.

Finally, two models of regression analyses were run, separately for men and women, in order to test for the relationship between pharyngeal presence of *S. aureus* and disgust sensitivity, when controlled for fT, cortisol, hsCRP level, and body adiposity. As participants’ age was not related neither with disgust sensitivity nor *S. aureus* pharyngeal colonization status, we did not control for age in the analyses.

Analyses were performed with Statistica 12.0 software (StatSoft Polska, Kraków, Poland). The results were significant at the *p* < 0.05 level.

## 3. Results

### 3.1. Descriptive Statistics

Descriptive statistics and sex differences in disgust sensitivity are presented in Table 2. Women exhibited higher total disgust sensitivity, which was mainly driven by the difference in sexual disgust sensitivity (Table 2).

Descriptive statistics of the controlled variables (cortisol, fT, hsCRP, and body adiposity) for men and women separately are presented in Table 3.

The results of Pearson correlation analysis showed that total (*r* = −0.09, *p* = 0.30, CI95% [−0.25; 0.98]), moral (*r* = 0.01, *p* = 0.88, CI95% [−0.16; 0.18]), and pathogen disgust sensitivity (*r* = 0.04, *p* = 0.64, CI95% [−0.13; 0.21]) were not correlated with fT level, but sexual disgust sensitivity was negatively correlated with fT in men (*r* = −0.23, *p* = 0.01, CI95% [−0.38; −0.06]). Cortisol level was not correlated with total (*r* = −0.03, *p* = 0.73, CI95% [−0.20; 0.14]), pathogen (*r* = −0.02, *p* = 0.82, CI95% [−0.19; 0.15]), or moral disgust sensitivity (*r* = 0.13, *p* = 0.14, CI95% [−0.04; 0.29]), but it was marginally, negatively correlated with sexual disgust sensitivity (*r* = −0.17, *p* = 0.052, CI95% [−0.33; −0.002]) in men. hsCRP level was neither correlated with total (*r* = 0.03, *p* = 0.73, CI95% [−0.14; 0.20]), pathogen (*r* = 0.04, *p* = 0.60, CI95% [−0.13; 0.21]), sexual (*r* = 0.05, *p* = 0.59, CI95% [−0.12; 0.22]), nor with moral disgust sensitivity (*r* = −0.03, *p* = 0.73, CI95% [−0.20; 0.14]). Body adiposity was not correlated with total (*r* = −0.01, *p* = 0.93, CI95% [−0.18; 0.16]), moral (*r* = −0.16, *p* = 0.06, CI95% [−0.32; 0.01]), sexual (*r* = 0.01, *p* = 0.86, CI95% [−0.16; 0.18]), and pathogen disgust sensitivity (*r* = 0.13, *p* = 0.12, CI95% [−0.04; 0.29]) in men.

The results of Pearson correlation analysis showed that in women, total (*r* = −0.08, *p* = 0.46, CI95% [−0.27; 0.12]), moral (*r* = 0.02, *p* = 0.85, CI95% [−0.18; 0.22]), sexual (*r* = −0.03, *p* = 0.74, CI95% [−0.23; 0.17]), and pathogen disgust sensitivity (*r* = −0.15, *p* = 0.14, CI95% [−0.34; 0.05]) were not correlated with fT level. Cortisol level was not correlated with total (*r* = −0.16, *p* = 0.12, CI95% [−0.35; 0.04]), moral (*r* = −0.10, *p* = 0.33, CI95% [−0.29; 0.10]), and pathogen disgust sensitivity (*r* = 0.05, *p* = 0.63, CI95% [−0.15; 0.25]) but was negatively correlated with sexual disgust sensitivity (*r* = −0.25, *p* = 0.01, CI95% [−0.43; −0.05]) in women. hsCRP level was not correlated with total (*r* = −0.06, *p* = 0.58, CI95% [−0.26; 0.14]), moral (*r* = −0.16, *p* = 0.13, CI95% [−0.35; 0.04]), sexual (*r* = 0.04, *p* = 0.72, CI95% [−0.16; 0.24]), and pathogen disgust sensitivity (*r* = −0.004, *p* = 0.97, CI95% [−0.20; 0.20]) in women. Body adiposity was not correlated with total (*r* = −0.10, *p* = 0.34, CI95% [−0.29; 0.10]), moral (*r* = −0.01, *p* = 0.92, CI95% [−0.21; 0.19]), sexual (*r* = −0.10, *p* = 0.31, CI95% [−0.29; 0.10]), and pathogen disgust sensitivity (*r* = −0.09, *p* = 0.39, CI95% [−0.28; 0.11]) in women.

The results of the chi-squared test showed that *S. aureus* was detected more often in women compared with men (X^2^(1) = 7.74, *p* = 0.005). A total of 47 men (vs. 90 without *S. aureus*) and 50 women (vs. 45 without *S. aureus*) were colonized with *S. aureus*.

### 3.2. Disgust Sensitivity and Pharyngeal Colonization by S. aureus

The results of t-test showed that the difference in disgust sensitivity between colonized and noncolonized individuals was significant only in men. Men colonized by *S. aureus* exhibited higher pathogen disgust (*p* = 0.04) compared to noncolonized men. The difference in sexual disgust sensitivity between individuals colonized and noncolonized by *S. aureus* was close to the level of statistical significance at *p* = 0.06 level, and colonized men exhibited higher sexual disgust sensitivity. There was no difference in moral disgust sensitivity between colonized and noncolonized men (Table 4).

The regression model showed that *S. aureus* colonization was only related with pathogen disgust in men but not in women and when controlled for adiposity, cortisol, fT, and hsCRP levels (Table 5).

## 4. Discussion

The results of our study showed that men colonized by *S. aureus* exhibited higher disgust sensitivity compared to noncolonized men. Women, in general, displayed higher disgust sensitivity compared to men, but there was no effect of *S. aureus* colonization status on the level of disgust sensitivity in women. It is also worth noting that the sex difference in disgust sensitivity was driven by the sexual disgust domain, whereas the difference observed for total disgust sensitivity in colonized and noncolonized men was mainly driven by the pathogen disgust domain. The difference in disgust sensitivity between colonized and noncolonized men was independent of immunomodulating hormones (testosterone and cortisol), inflammation markers (hsCRP), or body adiposity.

Endogenous *S. aureus* infection can be extremely invasive due to its ability to produce many virulence factors, such as adhesive proteins, surface molecules, capsular polysaccharides, and highly virulent toxins (toxic shock syndrome toxin, enterotoxin B, or Panton–Valentine leucocidin), enabling bacterial spread, host tissues invasion, and cells lysis [36]. Although in asymptomatic carriage state toxins expression is down regulated, during an active infection, toxins can be expressed in high quantities [37]. Heightened avoidance of potentially infectious agents, evoked by elevated pathogen disgust sensitivity in asymptomatic carriers, may help to reduce the risk of auto-infection development in order to minimize the possible harmful effects of enhanced toxins expression. Also, as disgust improves immune system effectiveness, inducing more aggressive response [3,18,19,20], heightened pathogen disgust sensitivity in individuals colonized by *S. aureus* may increase immune defenses, helping to maintain the balance between *S. aureus* virulence factors and host’s immune system effectiveness.

Sex difference in the relationship between pathogen disgust sensitivity and pharyngeal presence of *S. aureus* may be explained by sex differences in immunity. Successful colonization by *S. aureus* depends on many factors, including bacterial virulence (the ability to invade host tissues) as well as effectiveness of a host immune defence mechanisms. It is well documented that sex contributes to the shape of immune response, reflected by lower prevalence of autoimmune disease, higher susceptibility to infection, lower antibacterial immune defence, and lower immune response to vaccine in men compared to women [38,39]. Previous research has shown that the risk of many serious infections is higher in *S. aureus* colonized compared to noncolonized individuals [22,28]. Not only can the newly acquired pathogens initiate the dissemination process of commensal *S. aureus*, leading to transition of *S. aureus* from asymptomatic carriage to infection and causing serious diseases [40], but also the coinfections of *S. aureus* and other bacteria are often more virulent and more resistant to eradication with pharmacological treatment than infection with a single pathogen [41,42]. As men are more vulnerable to infections compared to women [38,39,43], they may also benefit more from heightened pathogen avoidance when being *S. aureus* carrier.

Sex differences in immunity are hypothesized to be related to immunosuppressive properties of testosterone [44]. However, in our study, testosterone level did not impact the relationship between pathogen disgust sensitivity and *S. aureus* colonization status neither in men nor in women. Additionally, in spite of the fact that cortisol level contributes to stress-induced infection susceptibility [34,45] and polymorphism in the glucocorticoid receptor gene influences the carrier state of *S. aureus* [31,46], similarly to testosterone level, no difference in cortisol level was found between individuals colonized and noncolonized with *S. aureus*, suggesting that genetics and other host factors are more important than hormone levels per se [47].

Women exhibited higher sexual disgust compared to men, but there was no difference in sexual disgust between women who were colonized and women who were not colonized with *S. aureus*. The difference in sexual disgust sensitivity between colonized and not colonized men was close to the statistical significance, suggesting that *S. aureus* carriage may also impact sexual disgust sensitivity only in men. Sexual avoidance, motivated by disgust, is somehow distinct from pathogen avoidance, with respect to the nature of the optimal avoidance behaviors, and also because assessing mate suitability and infection risk requires different sets of information [32]. Whereas pathogen detection relies on cues such as body fluids, foulness or mold, the assessment of mate suitability depends also on other cues—many of which are not relevant to proximal pathogen avoidance (e.g., avoidance of cues of possible incest). Thus, the shift in sexual disgust sensitivity may not be as pronounced as the shift in pathogen disgust domain in response to potentially infectious agents.

Additionally, the results of our study showed that sexual disgust sensitivity was negatively related with cortisol level (although for men the result was only marginally significant). Cortisol level has been shown to be negatively related with an individual’s biological condition and mate value [48], thus one may expect that individuals with higher cortisol level (i.e., less attractive) should exhibit lower sexual disgust sensitivity, enabling lower choosiness and sexual permissiveness, increasing chances for reproduction. In women sexual disgust was also negatively related to higher body adiposity and positively with hsCRP level, two factors that may be related to a woman’s lower attractiveness, perceived health, and mate value [48,49]. However, further investigation, using multiple hormonal measurements, is needed to verify the relationship between cortisol level and sexual disgust susceptibility in both sexes.

We found no relationship between pharyngeal presence of *S. aureus* and moral disgust sensitivity. Moral disgust pertains to social transgressions, including antisocial acts, such as lying, cheating, and stealing, that harm others directly and/or impose diffuse costs on one’s social group. Such behaviors inflict costs directly and they can disrupt cooperative relationships, social networks, and group cohesion [35]. Although all three disgust domains share neural correlates [50], moral disgust is elicited by different cues, has distinct personality correlates [51], and also motivates different behavioral strategy than pathogen and sexual disgust [35]. Whereas pathogen disgust is expected to motivate proximal avoidance of perceived infection risks and sexual disgust motivates avoidance of individuals within the specific context of sexual interactions, moral disgust should motivate avoidance of social relationships with norm-violating individuals [35] and thus might be not necessarily related to immune system functioning.

Finally, some limitations of our study need to be addressed. The main limitation is that only a single swab collection was used to determine *S. aureus* colonization status. Although there is no standard procedure defining how many swabs allow for carriage status determination, some longitudinal studies suggest that two consecutive positive result of *S. aureus* swabs, obtained within a few days interval, are needed to diagnose persistent carriage [52]. According to this, the “carriers” group in this study may comprise both intermittent and persistent *S. aureus* carriers. As persistent carriers have higher bacterial load [53], higher specific antibody titer [23], and higher risk of subsequent infections [28], compared to intermittent carriers, one may expect that these two groups may also differ in disgust sensitivity and should be analyzed separately. However, these assumptions would require further studies, as based on the results of this study and up-to-date literature, we are not able to explain the proximate mechanism of the relationship between pharyngeal presence of *S. aureus* and individual’s disgust sensitivity. It is not clear if specific antibody titer or higher bacterial load may impact a carrier’s prophylactic behavior or is there some other mechanism driving the relationship between *S. aureus* colonization and disgust sensitivity. Additionally, in further research it would be worth to attempt to characterize detected *S. aureus* strains in terms of virulence genes, that confer the ability to cause various disease, in order to differentiate potential pathogenicity of isolated strains and its relationship with an individual’s disgust sensitivity.

Furthermore, disgust sensitivity was only evaluated based on self-report questionnaire, which may not accurately reflect the real avoidance behavior. More distinct effects and stronger responses might be observed if individuals were confronted with more ecologically valid disgust elicitors, e.g., photos, movies, objects [4], or were interviewed on their real-life behaviors. Furthermore, apart from cortisol and testosterone, also other hormones, that were not controlled in this study (e.g., progesterone), may also impact an individual’s disgust sensitivity [5,6]. Although recent studies showed that changes in sex hormones levels in the menstrual cycle are not related with disgust sensitivity [7], it would be worth to control for estradiol and progesterone levels in future research. Additionally, this would allow to verify self-reported moment of the menstrual cycle. Finally, one may also presume that if latent pharyngeal presence of *S. aureus* may impact an individual’s disgust sensitivity, it is also possible that other latent pathogens (for instance Epstein–Barr virus, cytomegalovirus etc.), that were not included in this study, might also play a role in modulating disgust sensitivity. This should be verified in the future studies.

## 5. Conclusions

Our research provides support for prophylactic hypothesis, explaining inter-individual differences in disgust sensitivity by showing that men burdened with asymptomatic *S. aureus* exhibit higher pathogen disgust sensitivity compared with men without pharyngeal presence of *S. aureus*. The pathogen pressure does not evoke changes in moral and sexual disgust domain.

## Figures and Tables

**Table 1:** Study group demographic information (*N* = 232).

Demographic Characteristic	Frequency (*N*)
Gender	
Women	95
Men	137
Place of living	
Big city and suburbs	222
Small city	8
Village	2
Education	
University degree or further	212
Students	20
Medical worker	
Yes	30
No	202
Smoking status	
Yes	32
Occasionally	55
No	145

**Table 2:** Descriptive statistics and differences in disgust sensitivity between men (*N* = 137) and women (*N* = 95).

Variable	Men	Women	Difference
	M ± SD	M ± SD	
Pathogen disgust	26.67 ± 6.10	27.99 ± 6.08	t(230) = −1.62, *p* = 0.11
Sexual disgust	16.52 ± 6.39	23.45 ± 7.36	t(230) = −7.68, *p* < 0.0001
Moral disgust	30.31 ± 6.38	29.44 ± 6.69	t(230) = 1.00, *p* = 0.32
Disgust—total	73.50 ± 12.65	80.93 ± 13.83	t(230) = −4.23, *p* < 0.0001

**Table 3:** Descriptive statistics of the controlled variables for men (*N* = 137) and women (*N* = 95).

Variable	Men		Women	
	M ± SD	Range	M ± SD	Range
hsCRP (µg/mL)	0.90 ± 1.34	0.01–12.23	1.51 ± 2.36	0.00–11.44
fT (pg/mL)	25.93 ± 9.52	0.76–48.30	1.32 ± 2.21	0.39–21.58
Cortisol (ng/mL)	187.61 ± 78.8	38.01–524.85	176.05 ± 66.96	36.37–360.55
Body adiposity (%)	17.75 ± 6.23	4.70–36.00	25.50 ± 7.57	3.50–42.90

**Table 4:** Differences in disgust sensitivity between individuals colonized and noncolonized by *S. aureus*.

Variable	Men			Women		
	Colonized *N* = 47	Noncolonized *N* = 90	Difference	Colonized *N* = 50	Noncolonized *N* = 45	Difference
Pathogen disgust	**28.08 ± 6.47**	**25.93 ± 5.79**	**t(135) = −1.98** ***p* = 0.04**	28.34 ± 6.35	27.60 ± 5.80	t(93) = −0.59 *p* = 0.56
Sexual disgust	17.91 ± 7.40	15.79 ± 5.70	t(135) = −1.86 *p* = 0.06	24.18 ± 7.40	22.73 ± 7.31	t(93) = −1.96 *p* = 0.34
Moral disgust	31.23 ± 4.68	29.83 ± 7.08	t(135) = −1.22 *p* = 0.22	29.36 ± 7.20	29.53 ± 6.16	t(93) = −0.13 *p* = 0.90
Disgust—total	**77.23 ± 12.68**	**71.55 ± 12.25**	**t(135) = −2.54** ***p* = 0.01**	81.88 ± 14.45	79.87 ± 13.20	t(93) = −0.71 *p* = 0.48

Bolded values are significant.

**Table 5:** Regression results of the relationship between disgust sensitivity and *S. aureus* colonization status in men (*N* = 137) and women (*N* = 95), controlling for fT, cortisol, hsCRP, and body adiposity.

	Pathogen Disgust		Sexual Disgust		Moral Disgust		Total Disgust	
	**Men**							
	**β (*p*)**	**Model *p***	**β (*p*)**	**Model *p***	**β (*p*)**	**Model *p***	**β (*p*)**	**Model *p***
*S. aureus* colonization	**0.19 (0.03)**	**0.05**	0.16 (0.06)	0.06	0.09 (0.30)	0.17	0.22 (0.01)	0.08
Cortisol (pg/mL)	0.02 (0.82)		−0.16 (0.06)		0.06 (0.47)		−0.04 (0.61)	
Body adiposity (%)	0.16 (0.07)		0.01 (0.95)		−0.14 (0.11)		0.01 (0.91)	
*S. aureus* colonization	**0.19 (0.03)**	**0.05**	0.15 (0.08)	0.06	0.08 (0.34)	0.18	**0.21 (0.02)**	**0.05**
fT (pg/mL)	0.02 (0.80)		−0.18 (0.06)		−0.06 (0.53)		−0.11 (0.24)	
Body adiposity (%)	0.17 (0.08)		−0.05 (0.62)		−0.17 (0.07)		−0.03 (0.74)	
*S. aureus* colonization	**0.19 (0.03)**	**0.05**	0.16 (0.06)	0.29	0.09 (0.90)	0.21	0.21 (0.01)	0.08
hsCRP (pg/mL)	−0.07 (0.43)		−0.03 (0.71)		0.01 (0.90)		−0.05 (0.61)	
Body adiposity (%)	**0.18 (0.04)**		0.04 (0.62)		−0.15 (0.09)		0.03 (0.71)	
	**Women**							
	**β (*p*)**	**Model *p***	**β (*p*)**	**Model *p***	**β (*p*)**	**Model *p***	**β (*p*)**	**Model *p***
*S. aureus* colonization	0.05 (0.62)	0.77	0.13 (0.22)	**0.04**	−0.01 (0.97)	0.89	0.08 (0.40)	0.30
Cortisol (pg/mL)	0.04 (0.73)		**−0.25 (0.01)**		−0.08 (0.45)		−0.16 (0.13)	
Body adiposity (%)	−0.08 (0.43)		−0.13 (0.22)		−0.02 (0.85)		−0.16 (0.13)	
*S. aureus* colonization	0.04 (0.68)	0.13	0.19 (0.30)	0.57	−0.02 (0.88)	0.99	0.06 (0.57)	0.39
fT (pg/mL)	−0.23 (0.03)		−0.04 (0.69)		−0.03 (0.80)		−0.14 (0.21)	
Body adiposity (%)	−0.14 (0.18)		−0.11 (0.30)		−0.02 (0.87)		−0.14 (0.20)	
*S. aureus* colonization	0.05 (0.61)	0.78	0.05 (0.62)	**0.02**	−0.01 (0.88)	0.99	0.04 (0.68)	0.28
hsCRP (pg/mL)	0.03 (0.82)		**0.30 (0.01)**		0.01 (0.93)		0.18 (0.12)	
Body adiposity (%)	−0.10 (0.39)		**−0.22 (0.04)**		−0.01 (0.89)		−0.17 (0.14)	

Bolded values are significant.
